# Ion Channels in Inflammatory Processes: What Is Known and What Is Next?

**DOI:** 10.1155/2016/6245731

**Published:** 2016-02-23

**Authors:** Mauricio A. Retamal, Michael V. L. Bennett, Pablo Pelegrin, Ricardo Fernandez

**Affiliations:** ^1^Centro de Fisiología Celular e Integrativa, Clínica Alemana Universidad del Desarrollo, Santiago, Chile; ^2^Albert Einstein College of Medicine, NY, USA; ^3^Murcia's BioHealth Research Institute IMIB-Arrixaca, 30120 Murcia, Spain; ^4^Universidad de Los Lagos, Osorno, Chile

Inflammation is the primary response of the immune system to infection or tissue injury. Ion channels in different immune cells are very important for inflammatory processes. In this context, connexin (Cx) and pannexin (Panx) cell-cell channels and unapposed hemichannels and P2 receptors play central roles in the initiation and/or progression of inflammation in different tissues. Thus, cellular responses during inflammation can be initiated and/or enhanced by the opening of Cx and/or Panx hemichannels, which in turn allow the release of ATP and other metabolites to the extracellular media. These can act as “danger” signals to further propagate the original inflammatory response. Extracellular ATP can activate important intracellular signaling pathways through the activation of P2 receptors. However, it is yet not completely clear how these channels become active in these processes, how they interact, and how their pharmacological modulation may have a potential advantageous or beneficial therapeutic effect. In this special issue, M. Leo et al. show that agonists of TNFR-1 and TNFR-2 increase voltage gated sodium currents in cultured DRG neurons. These authors suggest that the activation of voltage gated sodium channels increases excitation of sensory neurons and this might explain the sensitization associated with neuropathic and inflammatory pain. On the other hand, kidneys are affected by ischemia, endotoxemia, and diabetic nephropathy, where the concentrations of proinflammatory cytokines increase. K^+^ channels play important roles in the function of renal tubule epithelial cells. Therefore, the effect of cytokines on K^+^ channels could be associated with alterations of tubular transport or onset of renal cell injury. Thus, K. Nakamura et al. provide an exhaustive review about the effect of proinflammatory cytokines on K^+^ channels present in the kidney. Ion channels are fundamental for normal lung function and their alteration may cause some lung diseases. Cl^−^ channels have particular relevance because they contribute to mucus synthesis, secretion, function, and mucociliary clearance. According to this idea, M. Sala-Rabanal et al. discuss the role of Cl^−^ channels, transporters, and regulators upon the progression of inflammatory diseases in lungs. Another type of channel that participates in lung function is the TRPA1 channel. A.-H. Lin et al. show that this channel is crucial for transducing ROS production in the extracellular space in Ca^2+^-dependent signaling pathways. This phenomenon could be important in our understanding of the relationship between cigarette smoke and lung inflammation. Finally, S. S. Kim reviewed several light-associated techniques useful for the study of P2X channels, which are quite important to several steps of inflammatory responses.

*Mauricio A. Retamal* *Mauricio A. Retamal* *Michael V. L. Bennett* *Michael V. L. Bennett* *Pablo Pelegrin* *Pablo Pelegrin* *Ricardo Fernandez* *Ricardo Fernandez*

